# Polyphenol-Rich Purple Corn Pericarp Extract Adversely Impacts Herbivore Growth and Development

**DOI:** 10.3390/insects11020098

**Published:** 2020-02-02

**Authors:** Mandeep Tayal, Pavel Somavat, Isabella Rodriguez, Tina Thomas, Bradley Christoffersen, Rupesh Kariyat

**Affiliations:** 1 Department of Biology, The University of Texas Rio Grande Valley, Edinburg, TX 78539, USA; mandeep.tayal01@utrgv.edu (M.T.); Bradley.christoffersen@utrgv.edu (B.C.); 2 School of Earth, Environmental, and Marine Sciences, The University of Texas Rio Grande Valley, Edinburg, TX 78539, USA; Pavel.somavat@utrgv.edu; 3 Mathematics and Science Academy, The University of Texas Rio Grande Valley, Edinburg, TX 78539, USA; isabella.rodriguez01@utrgv.edu; 4 Department of Chemistry, The University of Texas Rio Grande Valley, Edinburg, TX 78539, USA; tina.thomas@utrgv.edu

**Keywords:** purple corn, polyphenols, tobacco hornworm, hatching rate, mass gain, pupation

## Abstract

Plant secondary metabolites such as terpenes, phenolics, glycosides, and alkaloids play various functional roles including pigmentation, foliar and floral volatile synthesis, hormonal regulation, and direct and indirect defenses. Among these, phenolic compounds are commonly found in plants, but vary in the distribution of their specific compounds among plant families. Polyphenols, including anthocyanins and tannins, are widely distributed and have been well documented for their roles- primarily in plant pigmentation and also in plant defenses. However, commercialization of such compounds for use in insect pest management is severely hampered by expensive, inefficient, and time-consuming extraction protocols. Using a recently developed inexpensive and easy extraction method using the byproducts of pigmented (purple) corn processing, we examined whether the crude pericarp extract rich in polyphenols can affect the growth and development of tobacco hornworm (*Manduca sexta* L.) caterpillars. Our findings show that purple corn pericarp extract negatively affected *M. sexta* egg hatching and larval mass gain and prolonged developmental time compared to regular yellow corn extract or an artificial control diet. We also found that these effects were more severe during the early stages of caterpillar development. These results conclusively demonstrate that purple corn pericarp, an inexpensive by-product of the corn milling industry, is a valuable product with excellent potential as an insect antifeedant.

## 1. Introduction

Plants produce a wide range of secondary metabolites that serve a gamut of functions. These include their role as floral scent that attracts pollinators [1,2,3,4], foliar volatiles that act as attractants or repellants for herbivores and predators [5,6,7,8,9], defensive toxins against herbivory [10,11,12,13,14], and in pigmentation [15,16]. Among these functions, the role of secondary metabolites (e.g., plant volatiles) in insect-mediated species interactions (e.g., herbivory and pollination) have been well documented in various study systems, and these studies have collectively found that these interactions, either mutualistic or antagonistic, are affected by a wide range of factors, both abiotic and biotic in nature [17,18,19,20]. Moreover, secondary metabolites are also tightly regulated and species-specific, both in synthesis and emission [21,22,23]. 

In addition to the various organic volatile compounds and their well-documented effects, plants also produce phenolics, glycosides, and alkaloids [12,24]. These compounds, while ubiquitous in angiosperms, are also found to vary both qualitatively and quantitatively among various plant families [25]. For example, coumarins (1, 2-benzopyrones) are common in many dicotyledonous families, including the Apiaceae, Asteraceae, Fabaceae, Moraceae, Rosaceae, Rubiaceae, and Solanaceae, while catechol (1, 2-di-hydroxybenzene) and phloroglucinol (1,3,5-trihydroxybenzene) have been found in leaves of Gaultheria species (Ericaceae) and as a glucoside in the peel of various citrus fruits (Rutaceae) [26]. In general, these compounds play an important role in cell division, hormonal regulation, and various plant biochemical and physiological activities including protection from biotic and abiotic stresses [27,28,29]. Besides their benefits to the plants against biotic and abiotic stresses, they also impart color to plant tissues. Anthocyanins, a major group among these are vacuolar pigments (e.g., cyanidin, delphinidin, peonidin, petunidin, pelargonidin, and malvidin) primarily found in floral organs that impart red, blue, black, and purple hues [30]. Biosynthetically similar to anthocyanins, tannins constitute another group of polyphenols, synthesized via flavonoid pathways [31,32,33]. These are structurally (C6-C3-C6)n molecules consisting of two or more flavan-3-ols, majorly found as two structural forms of condensed tannins and hydrolysable tannins [34]. Although known for their potential defensive role against insect herbivores [33], there is a limited understanding of methods to commercially extract and use them as a biopesticide or herbivore deterrent.

Somavat et al. (2018) developed an efficient and economical methodology to isolate and quantify these compounds from purple corn pericarp, which is essentially a waste product of corn processing [35]. Besides flowers and fruits from various plant families rich in these compounds [16,36,37], colored corns (red and purple varieties) are also a rich source of polyphenols including anthocyanins and tannins [38,39]. The Andes region of Peru has been reported to be the origin of purple corn; however, they are also produced and consumed across Argentina, Bolivia, Ecuador, Mexico, Central America, and the United States [39]. Because of its rich purple pigments, it has long been used to color food and beverages [39,40,41]. Due to high biological activity of these compounds (anthocyanins in particular) as an antioxidant [42], anti-obesity [43,44], anti-cancer [42], and anti-inflammation compounds [45], it is plausible to expect that they may also have other effects, acting as an insect deterrent. The few studies that have explored their possible applications (primarily anthocyanins) as an anti-herbivore compound [15,46,47] have collectively found that these compounds protect plants through aposematic coloration and defensive mimicry, camouflage, and even as direct toxins [15,47,48]. However, none of these studies have used pericarp extracts to test its effectiveness against insect herbivores.

Continuing the long quest for identifying bioactive compounds with insecticidal/insect deterrent activity [49,50,51,52,53,54,55,56], we hypothesized that the pericarp extracts may also negatively affect herbivore growth and development. We tested this hypothesis using the tobacco hornworm caterpillar (*Manduca sexta L.*), a model herbivore for examining growth and development traits (easy to rear in lab with short lifecycle and distinct growth stages) [8,9,57,58]. We have previously used this insect model to disentangle the effects of direct and indirect physical and chemical plant defenses [7,9,56,57,58] on herbivore traits. Based on our previous work on economic recovery of polyphenol-rich extract from purple corn pericarp, we hypothesize that on account on its high polyphenolic concentration, this extract can be used as a natural pest deterrent against a number of insect species. However, we used *M. sexta L.* to demonstrate the proof of concept and plans are in place to expand these studies to include a number of other crop pests. Using the patent-pending extraction techniques elaborated in a coauthor’s earlier work [35,39,59,60,61], this extract can be economically prepared at a larger scale and can be deployed in a number of crops by spraying on plants. We first quantified the amount of these compounds in purple and yellow corn pericarp extracts, and then designed herbivore growth and development experiments with three treatments: (a) artificial diet enriched with purple corn pericarp extract, (b) artificial diet enriched with yellow corn pericarp extract as a positive control, and (c) an artificial control diet without any extract added as an additional control, to remove any extract effects. 

## 2. Materials and Methods

### 2.1. Corn Dry Milling for Pericarp Recovery

The dry milling protocol proposed by Rausch et al. (2009) was used with minor adaptation to recover purple and yellow corn pericarp [62]. An electronic moisture meter (GAC 2500-UGMA, DICKEY-john, Auburn, IL, USA) was used to estimate the corn moisture content prior to the processing. The corn kernels were then mixed with an appropriate amount of water and tempered in sealed 1 gallon plastic bottles rotated horizontally at 1 rpm for 20 min to allow for complete water absorption by the kernels. After tempering, the kernels were passed through a specially designed custom-made lab-scale horizontal drum degerminator. The degermed fractions were collected in two plastic boats and dried in a convection oven for 2 h at 49 °C. After conditioning, the degermed fractions were sifted on a 5-mesh screen for 3 min using a lab scale sifter (Model RX-812, WS Tyler, Mentor, OH, USA). 

**Figure 1:**
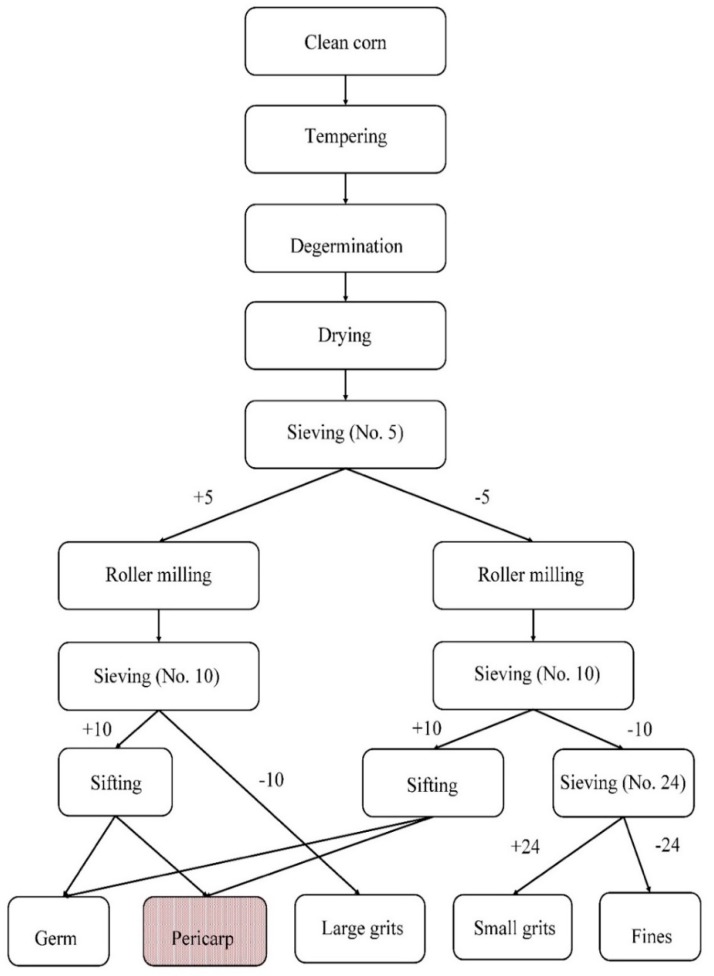
Process schematic of lab-scale dry milling protocol. The numerals represent the size of screen used, and + and – symbols represent the fractions recovered at the top and under those sieves, respectively (adapted from Rausch 2009) [64].

The fraction collected at the top of the screen (+5) and the material which passed through the screen (−5) were separated and roller milled twice using a lab scale roller mill (Micromill, Apollo Machine & Products Ltd., Saskatoon, Saskatchewan Canada). After a series of sieving procedures, the germ and pericarp collected from +5 and −5 fractions were sifted using a lab scale aspirator (Model 6DT4, Kice Industries, Wichita, KS, USA) to separate pericarp from the germ fraction, which is typically 10% (db) for purple corn [63] (Figure 1).

### 2.2. Pericarp Extract Preparation

Purple and yellow dent corn pericarp samples (5 g) recovered from dry milling were steeped in glass bottles containing 100 mL deionized water. These bottles were incubated at 52 °C for 24 h and stirred at 100 rpm to prepare liquid extracts [39]. After steeping, the extracts were centrifuged at 5000 rpm for 5 min, and the filtrate collected was incorporated in the artificial caterpillar diet.

### 2.3. Quantification of Anthocyanins, Tannins, and Total Polyphenols

The pH differential method was used to quantify the amount of total monomeric anthocyanins present in purple corn pericarp extract using a 96-well microplate reader (Multiskan Sky Microplate Spectrophotometer: #51119600, Thermo Fisher Scientific, Cambridge, MA, USA) in six replicates [62,63]. Dilution of pericarp extract (1:20) was made by taking 50 µL extract and 950 µL deionized water. The extract was mixed with two different buffers at different pH ranges (pH 1.0, 0.025 M KCl, and pH 4.5, 0.40 M sodium acetate). These diluted solutions at each pH were transferred (six replicates) to a 96-well plate, and absorbance was read at 520 and 700 nm. The concentration of total monomeric anthocyanins (mg/L) was calculated by using the following formula and reported as mg of cyanidin-3-O-glucoside (C3G) equivalent per kg pericarp:

Total monomeric anthocyanins (mg/L) = (A*MW*D*1,000)/(ɛ*0.45*PL)

where A = (A520 nm–A700 nm) at pH 1.0 – (A520 nm–A700 nm) at pH 4.5, MW is molecular weight, i.e., =449.2g/mol, D is the dilution factor, 1000 is the conversion factor from g to mg, ɛ is molar extinction coefficient for C3G = 26,900 L/mol, PL = 1 cm path length, and 0.45 was used as a conversion factor for adapting the established method to a plate reader.

Total condensed tannins were measured using the vanillin assay adapted from Burns (1971) [64]. Briefly, the extracts were diluted using methanol, and the reaction was carried out using a 1:1 ratio of 8% HCl: 1% vanillin. The absorbance was read at 500 nm in six replicates using a Multiskan Sky Microplate Spectrophotometer (Thermo Fisher Scientific). Catechin was used as a standard to report the results as mg catechin equivalent on a dry weight basis. A standard curve was determined by using a range of catechin standards. The tannin concentration was expressed as mg/kg pericarp.

The polyphenol concentration of purple and yellow corn pericarp extracts was also measured in six replicates employing Folin–Ciocalteu’s method adapted to a microassay as reported earlier [65]. Extracts were diluted (1:10) using deionized water, and 50 μL of diluted extract, standard, and blank were added to 125 μL of Folin–Ciocalteu’s phenol reagent. After 5 min, 750 μL of 20% Na_2_CO_3_ was added and the solution was allowed to react for 10 min. The absorbance was measured at 690 nm using a Multiskan Sky Microplate Spectrophotometer (Thermo Fischer Scientific). The polyphenol concentration was expressed as g of gallic acid/kg pericarp. 

### 2.4. Insect Colony

*M. sexta* eggs were bought from a commercial vendor (Great Lake Hornworm Ltd. Romeo, Michigan, USA) and were allowed to hatch in petri dishes (8.8 cm diameter, Mid Sci, Valley Park, MO, USA) on a moist filter paper in a growth chamber (16 h light/8 h dark, 25 °C day/22 °C night, 65% RH) [9]. We also have an active colony in the lab, and we periodically introgress with wild caught *M. sexta* (native to the region) to minimize any inbreeding effects. After hatching, the larvae were moved to plastic containers (35 cm long × 10 cm high × 15 cm wide) and reared on an artificial diet [9]. Newly molted larvae were used according to the experiment requirements. 

### 2.5. Artificial Diet

*M. sexta* caterpillars were allowed to feed on a wheat germ-based artificial diet prepared as per recommendations from the supplier (Frontier Scientific Services, Newark, DE, USA). We boiled 1000 mL of water to 85 °C, then added 200 g of artificial diet, and mixed thoroughly until there were no more lumps [9]. 

### 2.6. Plant Material

Tomato seeds (*Solanum lycopersicum*: Variety: Valley Girl), were bought from an online vendor (Johnny’s Selected Seeds, Winslow, ME, USA). Seeds were in sown in growth media (Sunshine professional growing mix: Sun Gro Horticulture Canada Ltd., Agawam, MA, USA) filled in the plastic trays (51 cm × 25 cm) and covered with thin transparent film to maintain optimum temperature of 27 °C for germination. At 2–4 leaf stage, the seedlings were transplanted individually to bigger pots (15 cm diameter) and kept in a green house at 25 °C and 65% RH. Plant nutrient requirements were met by applying OMRI (Organic Material Review Institute, Eugene, OR, USA)-listed diluted organic fish emulsion fertilizer (NPK 5:1:1, Alaska Fish Fertilizer, Pennington Seed, Inc., Madison, GA, USA) at 3 mL per pot once every two weeks. Plant growth and health was maintained regularly until plants were ready for experiment.

### 2.7. Detailed Experimental Design

We used three diets differing by either the presence or absence of pericarp extracts. Out of three groups, one diet group without any pericarp extract was used as a control while the other two diets contained purple corn and yellow dent corn pericarp extracts. Purple corn and yellow corn pericarp extracts were prepared by steeping 5 g pericarp of each corn type in 100 mL of water (5 g/100 mL), respectively, while control diet was prepared without adding any extract. Initially, to study dose-dependent effects, the experiment was started with three concentrations (5, 3, and 2 g) of purple corn pericarp extract but were then pooled since there was no detectable differences among the three concentrations. In order to prepare each treatment diet, we boiled 1000 mL of water to 85 °C, then added 200 g of artificial diet (Frontier Scientific Services) and mixed them thoroughly. During mixing, we added 1 g of agar and 40 mL of respective pericarp extracts. The diet was allowed to cool to 45 °C before adding the extracts and mixed thoroughly until there were no lumps. The diet was allowed to solidify and then made into ~1 cm^3^ cubes and used for the experiments.

### 2.8. Bioassays

All diet-based caterpillar assays were conducted in lab at 27 °C and 65% RH. To begin with, diets were allocated separately to different petri dishes comprising respective treatments. For each individual petri dish, diet was cut into ~1 cm^3^ cubes and placed in corresponding petri dishes and labeled by treatment and date. Different caterpillar growth and development parameters examined are as explained below:

#### 2.8.1. Egg Hatching

To test the effect of purple corn pericarp extract on egg hatching, 2-day-old viable eggs were kept on top of a ~1 cm^3^ cube of diet in different petri dishes. After 24 h, the eggs were examined for hatching percentage. The number of hatched eggs was recorded after 48 and 72 h. Each treatment had a sample size of 30.

#### 2.8.2. First Instar Survival

Newly hatched first instar caterpillars (N = 15) were allowed to feed on three treatment diets. After 24 h, the larval movement was used as an index to determine if the larvae were dead or alive. We recorded the number of alive larvae for three days until they became second instar. 

#### 2.8.3. Mass

Twenty-five (N = 25) first instar caterpillars were pre-weighed and placed on diets per treatment, and then caterpillar weight was recorded once every other day until they had reached the fifth instar larval stage and were ready for pupation. Additional data on caterpillar survival throughout the larval stages was also collected.

#### 2.8.4. Mass Gain

Using the following equation, we calculated two types of mass gain: (i) instar-specific relative mass gain over previous mass and (ii) final relative mass gain over initial mass.

$$\mathrm{Mass}\mathrm{gain}=\frac{\left(\mathrm{Final}\;\mathrm{mass}-\mathrm{Initial}\;\mathrm{mass}\right)}{\mathrm{Initial}\;\mathrm{mass}}$$

#### 2.8.5. Feeding Behavior

Apart from examining larval mass, we also examined their diet feeding preference. For this, on every other day just before the mass measurements, we observed if the caterpillars were feeding on the diet pellet or not. The caterpillars feeding on diet were recorded as on diet and others as off the diet. 

#### 2.8.6. Ethovision

To examine in detail whether the caterpillars were able to distinguish two treatment diets (purple corn pericarp and artificial control) based on color, odor, or texture. We also ran a behavioral assay experiment using the automatic video tracking Noldus Ethovision set-up (EthoVision XT 14: Noldus Information Technology, Wageningen, The Netherlands) mounted on an open field-testing arena. The arena (75 × 60 cm) was made up of wood (Thermo Fisher Scientific) with the bottom side covered with paper sheet from the inside to avoid any friction. In this experiment, 1 cm^3^ cubes of each diet was kept 10 cm apart in the center of an arena. Each trial consisted of 4 h starved, two newly molted third instar caterpillars (reared on artificial control diet) released at an equal distance (43 cm) away from diet cubes. We recorded their movement and behavior parameters for 1 h. These parameters included total distance (cm) travelled by each caterpillar, their velocity (cm/s), and feeding preferences. After every trial, the whole arena was wiped with ethanol (50% v/w) and diet positions were interchanged. We ran a total of 10 trials with 20 caterpillars in both treatments.

#### 2.8.7. Time to Pupate

Once the larvae reached fifth instar and started to clearly show the dark pulsating vein on the dorsal side (see Supplementary video S6), stopped feeding, and were wandering in the petri dishes, larvae were transferred to a plastic container with wood shavings (Natural Aspen small animal bedding: Petco Animal Supplies, Inc., San Diego, CA, USA) to provide them a dark environment to undergo pupation. Time to pupate was recorded for each caterpillar counting from the date of first instar stage to the date when they reached pupation (collected every day in the morning).

#### 2.8.8. Diet Switch Assay 

In addition to the mass-based assays with three treatments, we also ran a diet switch assay. For this, 45 first instar *M. sexta* caterpillars were pre-weighed, and 30 of them were placed on artificial control diet and other 15 on a purple corn extract diet, and they were allowed to feed and develop. After they molted to third instar, we removed 15 caterpillars from control diet, weighed and transferred them to purple corn extract diet, and moved all 15 caterpillars from purple corn extract diet back to control diet. The three new diet treatments (control, control moved to purple corn extract diet, and purple corn extract diet moved to control diet) were again continuously monitored and their mass measured at regular intervals. The reasoning for this experiment was to test whether the effects of purple corn diet, if any, could be compensated by switching them to regular diet or vice versa. Ideally, we should have had additional treatments with different growth stages for the switch experiment, but logistical constraints prevented us from doing so.

#### 2.8.9. Spray Experiment

To test whether pericarp extract has any insecticidal properties against *M. sexta* larvae when applied on plants, we conducted a spray experiment in which tomato plants were sprayed twice with different pericarp extracts. The experimental design consisted of three treatments including four plants per treatment fed with two caterpillars and 50 mL spray on each plant (N = 8) of (i) 4% diluted purple corn extract (v/v), (ii) 4% diluted yellow corn extract (v/v), and (iii) water (control) followed by mass gain and frass mass quantification. To start with, 4-week-old tomato plants were fully sprayed from all directions with treatment solutions at least 24 h before starting the experiment. We placed two pre-weighed third instar caterpillars per plant covered with mesh bags (15 × 25 cm: Kinglake fruit protection bag; Amazon.com) comprising a total of eight caterpillars per treatment. After 4 days, the caterpillars were removed from plants and weighed again to calculate mass gain. Caterpillar frass was calculated by weighing the total frass collected in individual mesh bags.

#### 2.8.10. Data Analysis

Due to the non-normal nature of some of our data sets, we used a combination of one-way ANOVA and nonparametric tests (Kruskal–Wallis test) followed by post hoc pairwise comparisons (Tukey and Dunn’s Test) to analyze most of our data. For anthocyanins, polyphenols, and tannin analyses, we used Mann–Whitney U tests (comparing purple corn and yellow corn). In growth assays, larval diet treatments were the factors, whereas caterpillar egg hatching, survival, caterpillar mass, mass gain, time to pupation, feeding preference, and frass mass were response variables. Separate analyses for caterpillar mass and mass gain were also carried out at each point of their development (nine larval mass data and eight larval mass gain data). On the other hand, feeding preference (on/off) data was analyzed using a chi-square (χ^2^) test [9]. For Ethovision experiments for caterpillar movement and feeding behavior, the distance and velocity data were analyzed using an unpaired *t*-test. All the data sets were analyzed using the statistical softwares JMP (SAS institute, Cary, NC, USA) and GraphPad PRISM (La Jolla, CA, USA). Specific test details are presented in supplementary tables.

## 3. Results

### 3.1. Quantification of Anthocyanins, Tannins, and Total Polyphenols

**Figure 2:**
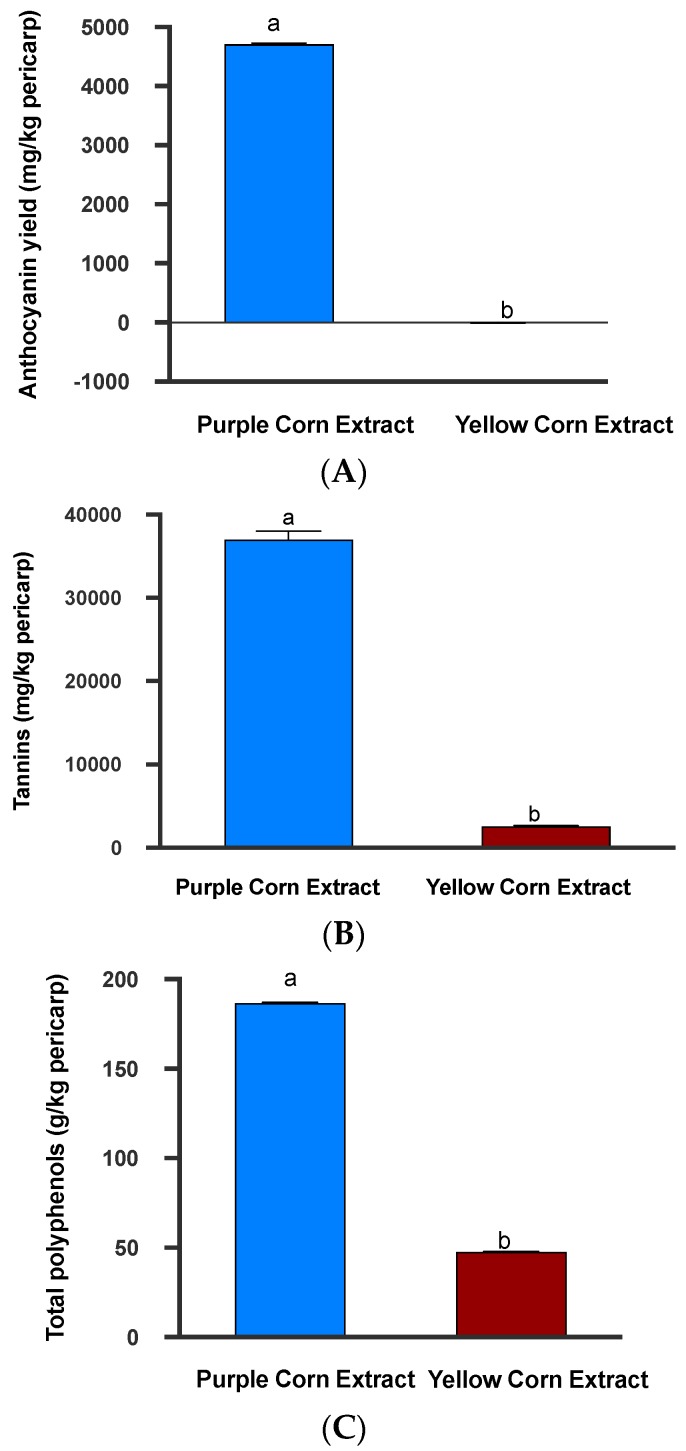
Mean anthocyanins (**A**), tannins (**B**), and total polyphenols (**C**) present in the purple corn and yellow corn pericarp extracts. Means followed by different letters are significantly different (Mann–Whitney U test, unpaired *t*-test, *p* < 0.05).

Based on quantification methods described above, we found that purple corn pericarp extract contained significantly higher concentrations of anthocyanins (mean ± SE: 4710.08 ± 43.13 mg C3G equivalent/kg pericarp; (Mann–Whitney U test, *p* = 0.0022; Figure 2A) and total polyphenols (mean ± SE: 186.59 ± 1.03 g gallic acid equivalent/kg pericarp; Mann–Whitney U Test, *p* = 0.0022; Figure 2B). In addition, no anthocyanins were detected in yellow corn pericarp extract (mean ± SE: −5.69 ± 4.23 mg C3G equivalent/kg pericarp) (Figure 2A). The results for anthocyanins quantified in yellow corn extract were negative. There were no anthocyanins present in yellow dent corn extract and since the spectrophotometric method for the quantification of anthocyanins utilized absorbance differences at two different wavelengths, these results came out with negative numbers. Results of tannin quantification also followed similar trends. Purple corn extract had more than 14 times the amount of tannins (mean ± SE; 36,964.23 ± 9879.10 mg catechin equivalent/kg pericarp) compared to yellow corn extract (mean ± SE; 2573.30 ± 687.74; Mann–Whitney U Test, *p* < 0.0001; Figure 2B). Collectively, it was clear that purple corn pericarp extract contained higher amounts of phenolic compounds compared to yellow corn extract. Total polyphenol concentration of pericarp extract from yellow dent corn was four times lower compared to purple corn pericarp (mean ± SE: 47.77 ± 0.58 g gallic acid equivalent/kg pericarp) (Figure 2C). 

A total of 40 mL purple corn pericarp and yellow corn pericarp extracts were mixed in respective insect diets. The diet prepared from purple corn extract contained approximately 0.94 mg C3G equivalent of anthocyanins, 373.18 mg gallic acid equivalent of polyphenols, and 73.92 mg catechin equivalent of tannins. On the other hand, the diet prepared from yellow corn pericarp extract contained 95.54 mg gallic acid equivalent of poyphenols, 5.14 mg catechin equivalent of tannins, and no anthocyanins.

### 3.2. Bioassays

#### 3.2.1. Effects of Purple Corn Pericarp Extract on Egg Hatching and First Instar Mortality

**Figure 3:**
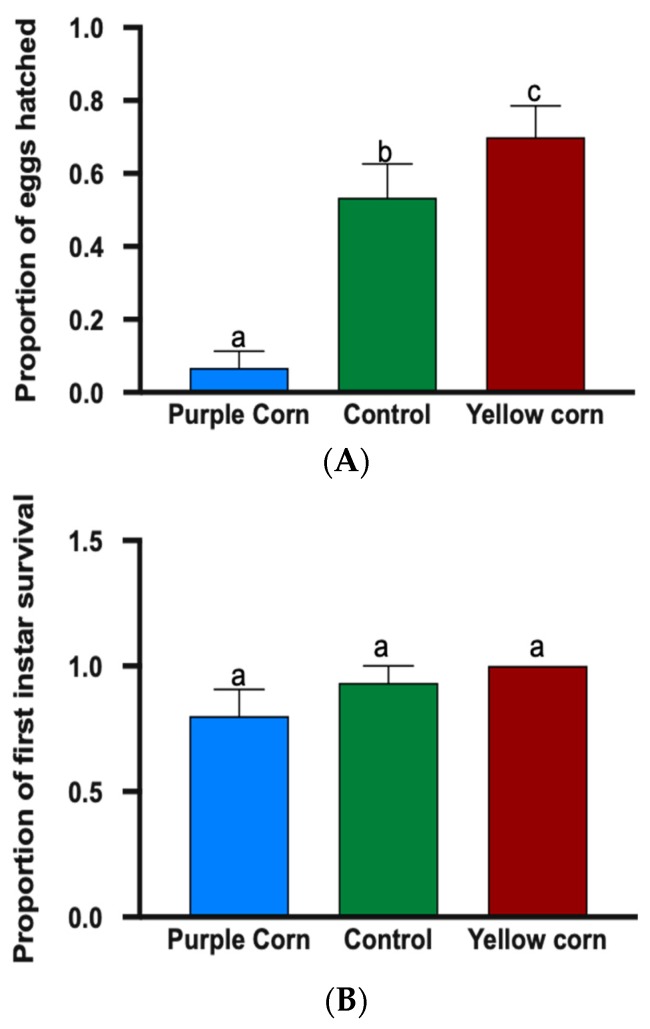
Proportion of eggs hatched (**A**) and mean first instar survival (**B**) among different treatments. Means followed by different letters are significantly different (Kruskal–Wallis tests, Dunn’s multiple comparison test, *p* < 0.05).

We found that egg hatching rate on diet containing purple corn pericarp extract was significantly lower (F value = 26.04, *p* < 0.0001) relative to other treatments (Figure 3A), as only 6% of eggs hatched on purple corn extract diet whereas yellow corn and control diet had egg hatching of 70% and 54%, respectively. However, when first instar caterpillars were allowed to feed, we did not find any significant difference (F value = 6.286, *p* = 0.1529) in the mean number of first instar caterpillar survival on different treatment diets, implying that there was no significant effect of pericarp extract on first instar mortality (Supplemantary Table S1) (Figure 3B).

#### 3.2.2. Effects of Purple Corn Pericarp Extract on Larval Mass

**Figure 4:**
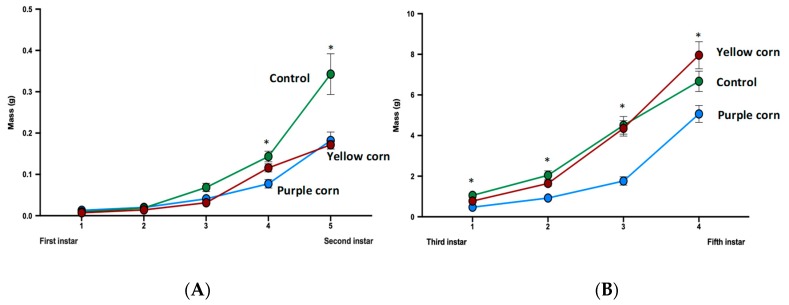
Mean first instar to second instar larval mass (**A**) and third instar to fifth instar larval mass (**B**) among different treatments (due to huge variation of larval mass scale from first instar to fifth instar, we split larval mass analysis into two parts). Means with an asterisk above indicate a significant difference between treatments (Kruskal–Wallis tests, Dunn’s multiple comparison test, *p* < 0.05).

We recorded larval mass once every other day until caterpillars reached fifth instar for a total of nine mass measurements throughout the whole larval stage. Except for the first mass (prior to being placed on diet treatments), we found significantly lower larval mass throughout the whole larval stage for caterpillars feeding on purple corn extract diet compared to yellow corn extract and control diet (Figure 4A,B). Statistics with individual test details are displayed in Supplementary Table S2.

#### 3.2.3. Effects of Purple Corn Pericarp Extract on Larval Mass Gain

**Figure 5:**
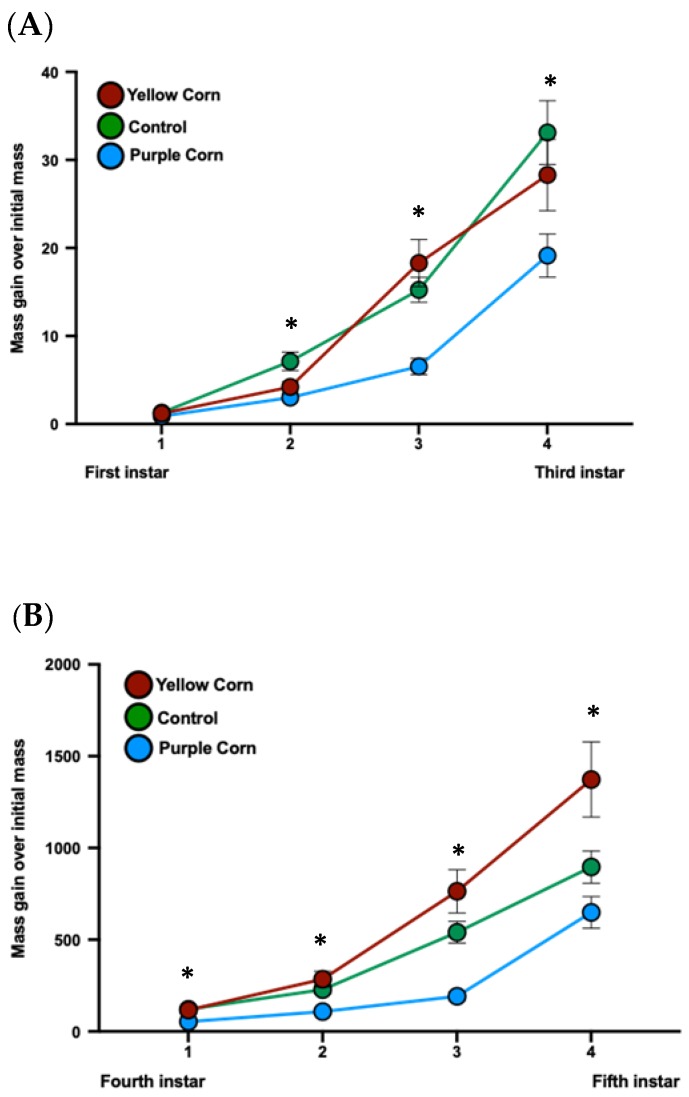

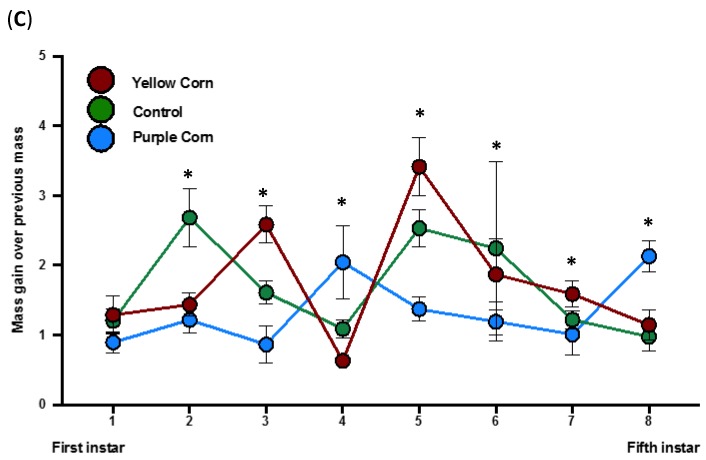
Mean larval mass gain from first instar to third instar (**A**) and mass gain from third instar to fifth instar (**B**) among different treatments. Due to huge variation of larval mass scale from first instar to fifth instar, we split larval mass analysis into two parts. Mean instar-specifc larval mass gain from first instar to fifth instar stage (**C**). Means with an asterisk above indicate a significant difference between treatments (Kruskal–Wallis tests, Dunn’s multiple comparison test, *p* < 0.05).

We calculated a total of eight mass gains (mass gain over previous mass and mass gain over first instar) through the end of larval stages of *M. sexta*. A significant negative impact of purple corn pericarp extract on mass gains compared to yellow corn extract and control diet was detected consistently until the fifth instar (Supplementary Tables S3 and S4) (Figure 5A,B). However, purple corn pericarp extract-fed fifth instar caterpillars gained significantly more mass (*p* = 0.0004) compared to individuals fed on control diet (Figure 5C), an interesting pattern that was not observed in any other measurements.

#### 3.2.4. Effects of Purple Corn Pericarp Extract on Larval Feeding Preference

Starting from first instar to fifth instar larval stage, we also recorded a total of eight feeding preference observations, one on every other day. For this, we examined caterpillar position in the petri plate (on/off the diet pellet). We found that a significant number (seven out of eight observations) of purple corn pericarp extract-fed caterpillars stayed away from the diet compared to those on either yellow corn extract and control diets or both, implying that purple corn pericarp extract was possibly the least preferred when compared to others (Supplementary Table S5 and Figure S1). 

#### 3.2.5. Ethovision

In Ethovision behavior tracking experiments, there was no difference in velocity (t = 0.36105, *p* = 0.5492) and total distance (t = 0.3063, *p* = 0.7629) travelled by larvae towards any diet (purple vs. control). However, when we visually analyzed the video of each trial, we observed that while caterpillars initially showed no preference of control vs. purple corn pericarp extract diet, most of them subsequently ate only control diet and stayed away from the purple corn extract (see Supplementary Videos S7). Among the 20 caterpillars tested, this behavior was observed in 14 of them (70%). We have previously documented that *M. sexta* host (food choice) is primarily through volatiles, and the lack of such homing behavior, [8] but reduced feeding on purple corn extract suggests antifeedant properties of the extract.

#### 3.2.6. Effects of Purple Corn Pericarp Extract on Caterpillar Survival Throughout the Larval Stages

In addition to monitoring caterpillar survival in the first instar, we also monitored *M. sexta* for mortality on the different treatments until they pupated. We found that while there was a treatment effect on caterpillar survival (F = 6.557, *p* = 0.03) (Supplementary Table S1), none of the pairwise comparisons were statistically significant. However, the percentage survival data clearly shows a trend where caterpillars reared on purple corn extract had the lowest proportion survived (77.3%) when compared to control (90.0%) and yellow corn diets (93.3%) (Supplementary Table S5 and Figure S2).

#### 3.2.7. Effects of Purple Corn Pericarp Extract on Pupation Time

**Figure 6:**
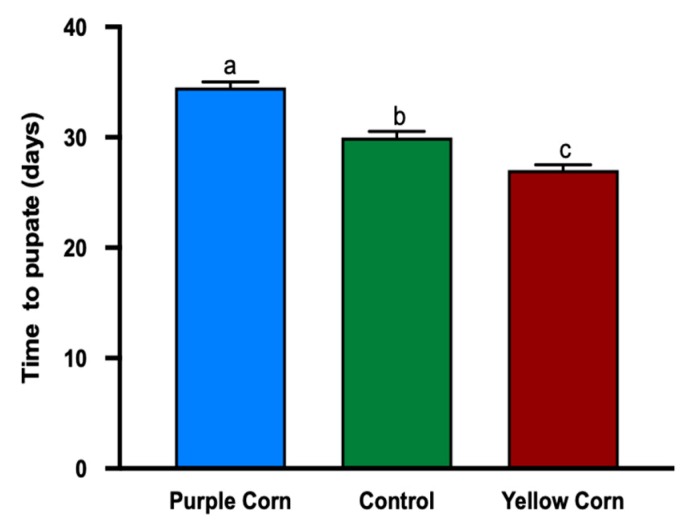
Mean time (days) to pupate of *M. sexta* caterpillars among different treatments. Means followed by different letters are statistically significantly different (Kruskal–Wallis tests, Dunn’s multiple comparison test, *p* < 0.05).

While examining the effect of purple corn pericarp extract on pupation time, we found that purple pericarp-fed larvae took significantly longer to pupate (number of days; F value = 50.91, *p* < 0.0001), when compared to yellow corn extract and control diet fed caterpillars. The pupation time in purple pericarp extract fed caterpillars was around 34 days, whereas control and yellow corn extract fed caterpillars had an average of 30 and 27 days, respectively (Figure 6).

#### 3.2.8. *M. sexta* Mass on Switched Diet Experiment

**Figure 7:**
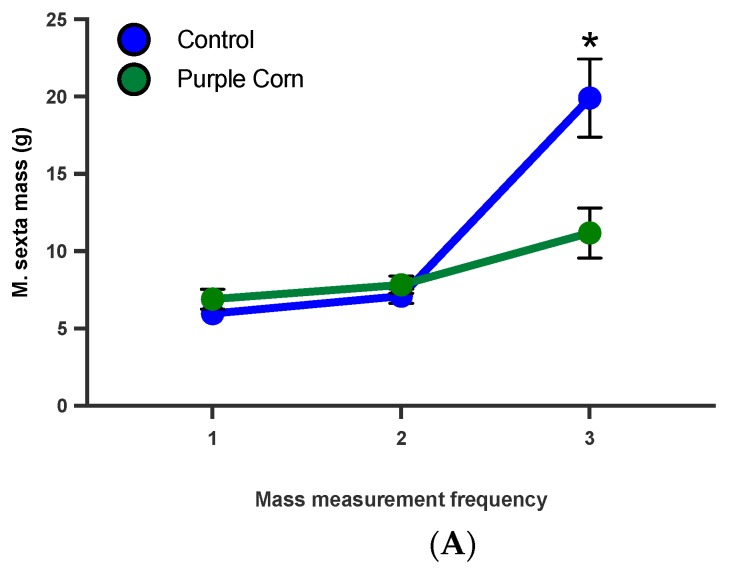

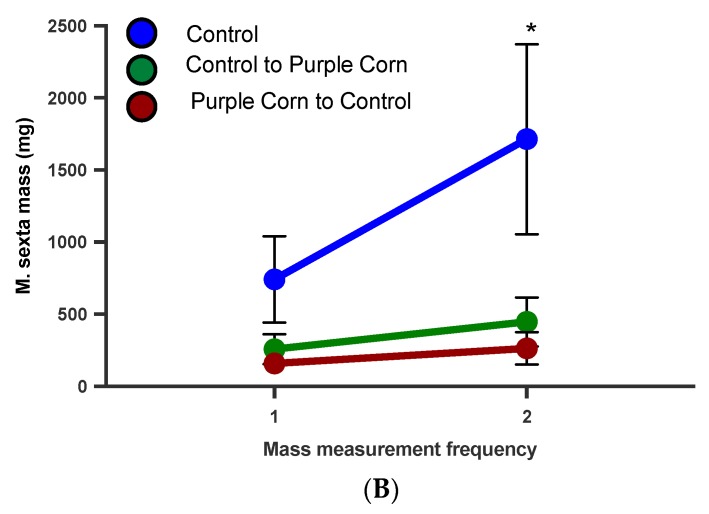
Mean larval mass before diet switch (**A**) and after diet switch (**B**) among different treatments. Groups with asterisks are statistical significant one-way ANOVA, unpaired *t*-test, Tukey’s HSD test, *p* < 0.05).

From our caterpillar mass data, it was clear that feeding on purple corn extract diet affected caterpillar mass (t = 2.226, *p* = 0.0329), although they had similar mass at the start of the experiment (Figure 7A). To disentangle whether these affects are dependent on larval stage, we ran a diet switch experiment. When the diets were switched (moving control fed to purple corn extract and viceversa), we found that control diet fed caterpillars continued to gain more mass compared to caterpillars switched to purple corn extract diet from control diet (Figure 7B). More importantly, we found that caterpillars that started on purple corn extract failed to gain comparable mass even after switching to control diet, clearly showing that early exposure to the extract diet severely affected their mass gain ability (F value = 4.740, *p* = 0.0226; Supplementary Table S7) (Figure 7B). 

#### 3.2.9. Effects of Plant-Sprayed Purple Corn Pericarp Extract on Larval Mass Gains and Frass Production

**Figure 8:**
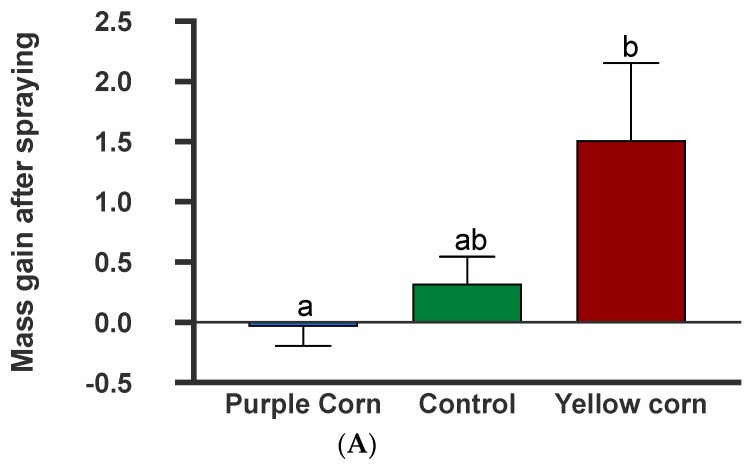

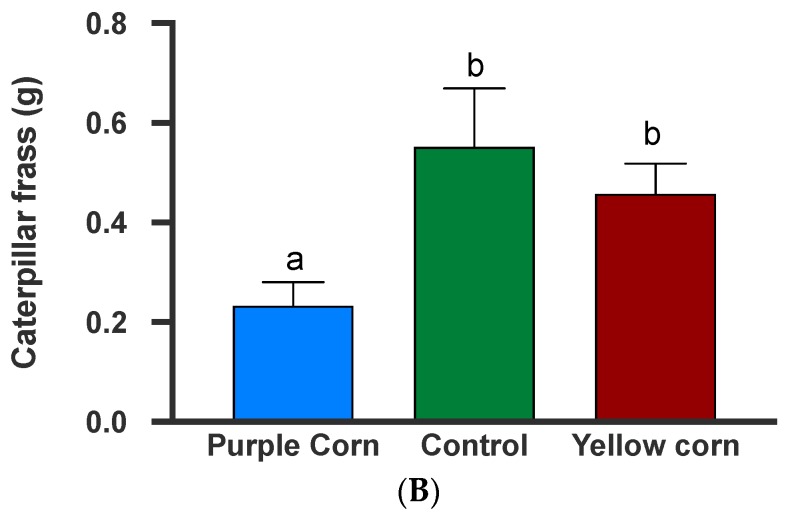
Mean larval mass gain after spray (**A**) and larval frass collected after two sprays (**B**) among different treatments. Means followed by same letters are not significantly different (Kruskal–Wallis tests, Dunn’s multiple comparison test, *p* < 0.05).

The results from the larval mass gain after spraying tomato plants with the extracts showed that spraying significantly impacted caterpillar mass gain (F = 10.54; *p* = 0.0051) (Supplementary Table S1). However, post hoc tests showed that while *M. sexta* larvae gained significantly lower mass on puple corn extract-sprayed tomato plants when compared to yellow corn extract (*p* = 0.0036), there were no differences among other pairwise combinations (Figure 8A). In addition to larval mass, we also collected caterpillar frass which also showed significant treatment effects (F = 5.414, *p* = 0.014) (Supplementary Table S1). Pairwise comparisons showed that caterpillars produced a lower amount of frass on purple corn extract-sprayed plants compared to control (*p* = 0.02) and yellow corn extract (*p* = 0.04), while there was no difference in frass mass between yellow and control spray (Figure 8B). Collectively, these data clearly show that the extracts retain their anti-feedant properties (lower mass gain and lower frass production) even after being sprayed externally on another host plant.

## 4. Discussion

Here, we have shown for the first time that a waste product of purple corn processing has immense potential as an anti-feedant against a damaging herbivore. Our results demonstrate the negative effects of anthocyanin/polyphenol-rich purple corn pericarp extract on the growth and development of *M. sexta* caterpillars. While our results cannot decisively disentangle the mechanistic underpinnings of these effects, they collectively suggest that the negative effects were likely due to combination of antifeedant properties of the extracts as well as the physiological stress imposed by polyphenols. We tested the effects of pericarp extract comprehensively throughout the larval stages including egg hatching, first instar survival, feeding preference, mass, mass gain, larval survival, and time to pupation. As eggs are the earliest life stage on which subsequent stages depend, low egg hatching percentage (6%) due to diet enriched with purple corn pericarp extract indicates its importance to limit subsequent larval growth and development (Figure 3A). This could possibly be due to developmental abnormalities induced by phenolic compounds present in diet, as previously documented in plant-based diet experiments [66]. 

Interestingly, we did not find any significant difference on first instar percent survival on different diets (Figure 3B). A possible explanation for neonate survivorship but high egg mortality might be increased metabolic enzyme production in early instars induced by secondary metabolites present in the diet. For example, it has previously been reported that consumption of phenolic glycosides led to increased activity of esterase enzymes in gypsy moth (*Lymantria dispar*) neonates, resulting in higher survival rates [67,68]. Moreover, the first instar larvae of *P. glaucus* were not affected by the neolignans (4,4’-diallyl-2’3’-dihydroxybiphenyl ether: a biphenyl ether) isolated from magnolia (*Magnolia virginiana*) hosts [68,69]. However, these effects were transient, and once the caterpillars molted to second and following instars, the effects were clear and severe.

Similar to the results reported by Datta et al. (2019) in tobacco cutworm (*Spodoptera litura*) caterpillars with Greater galangal (*Alpinia galanga*) extracts, we found that *M. sexta* larvae fed on purple corn pericarp extract-containing diet had lower mass and mass gain in early larval stages (Figure S4A,B and Figure 5A,B) [68]. The low mass and mass gain might be due to either the negative impact of pericarp extract ingested by them through diet or its antifeedant properties [2,70,71,72,73,74]. To confirm this, we did a no choice (on/off) and a choice assay using Ethovision behavior tracking system. While we found that there were no differences in caterpillar movement and choice (velocity and the distance travelled) between purple corn pericarp extract and control diet, they preferred to not feed on purple corn extract diet. However, analyzing video recordings (see Supplementary Videos S7) collected during the Ethovision experiment, it was clear that the caterpillars showed a preference for the control diet, very similar to the on/off assay results (Supplementary Figure S1). Significant reduction in larval mass in response to nutritional stress is speculated to negatively affect their adult fitness and reproduction. In addition, it is also well understood that low-quality host food can result in decreased immune response in *M. sexta* larvae, making them more susceptible to pathogens [75]. In line with these results, we did find a trend with caterpillar survival, where only 70% of the caterpillars on purple corn pericarp extract diet were able to successfully pupate while other diets had more than 90%. However, whether this increased mortality is due to compromised immune response needs to be investigated.

Contradictory to all other mass gain data, we found significant increase in mass gain of fifth instar on pericarp extract (Figure 5C) possibly due to a compensatory feeding response to stress by low quality and toxic food. As previous studies have shown that nutritionally stressed caterpillars make some physiological adjustments, i.e., producing detoxifying enzymes [76,77] or altering efficient food use [78,79,80], we speculate that similar mechanisms might have resulted in rapid growth of late instar caterpillars. However, the possible toxicity encountered during the early stages significantly derails their progress in mass gain and other traits, even after compensatory feeding in later stages. This was even more pronounced in the switch diet where the caterpillars failed to “catch up” even when moved to control diet after fed on purple corn extract until third instar. These findings are consistent with van Dam et al. (2000) who found the compensatory feeding and increased larval mass in *M. sexta* after shifting from Methyl jasmonate (MeJA; a phytohormone compound involved in plant defenses)-treated plants to untreated control plants [81]. 

Adding to the compensatory growth, we also found that larvae fed on pericarp extract took significantly longer time to pupate (Figure 6) compared to other treatments, suggesting developmental delay. Previous studies have reported similar effects of toxic diets. For example, Blue Myrtle-Cactus (*Myrtillocactus geometrizans*) extracts (rich in sterols and triterpenoids) delayed pupation and molting in fall armyworm (*Spodoptera frugiperda*) [82,83,84]. A possible explanation for the increased developmental time (time to pupate) may also be due to midgut phenoloxidase inhibtion and molting sclerotization toxicity, caused by phenolics [83]. It is well understood that resource acquisition in larval stage plays a critical role in an individual’s adult stage and henceforth in life history and fitness [85,86]. Based on our data, we speculate that pericarp extract might have potential cascading effects on pupal mass, adult fitness, and dispersal as well as their mating success, an area we are currently exploring.

In the spray experiment with tomato plants, we only found significant differences in caterpillar mass between purple corn and yellow corn treatment, but not with control (Figure 8A), While the trend is encouraging, lower extract solution concentration (4%) might be one reason for the lack of a significant difference. Further, since the artificial diet is highly concentrated and directly ingested by the larvae, whereas in plants, these low concentration extracts might not stick to the plant and not be ingested in sufficient amounts to be effective. Future studies could explore dose-dependent effects of pericarp extract against *M. sexta* larvae. Interestingly, we did find that the caterpillars on purple corn diet produced less frass, compared to both control and yellow corn diet, suggesting either starvation or lower digestibility on purple corn-sprayed plants, reinforcing their negative impacts.

While our extract quantification clearly showed dramatic differences for polyphenols between purple corn extract and yellow corn extract, we did not quantify any other toxins (and non-toxins) possibly present in the pericarp. While our experiments have controls with yellow corn extract and no-pericarp added regular diet somewhat mitigates this concern, a detailed analytical chemistry-based approach is needed to quantify all possible biologically active compounds in the pericarp. In addition, since purple corn contains anthocyanins and a number of other polyphenolic compounds, such as tannins, phenolic acids, and flavonoids [87], more research is needed to study whether the effects on life history traits are due to an individual compound (e.g., tannins) or due to a synergistic effect of these compounds. Different compounds present in the extract should be isolated and their dose-dependent effects should be individually studied in all possible combinations. Additionally, the ability of herbivores to exhibit plasticity in response to diet quality variations gives us directions to study if pericarp extract affects integrated compensatory responses of insect herbivores [88,89]. Additionally, apart from lepidopteran pests, it will be interesting to test the effects of the extract against pests with different feeding habits, such as aphids or white flies. Future studies are also required for a better understanding of the underlying mechanisms and mode of action of polyphenol-rich purple corn pericarp extract at the molecular level—some of the other areas we are currently exploring.

## 5. Conclusions

We concluded that purple corn pericarp extract rich in polyphenols has severe negative effects on growth and development of tobacco hornworm (*M. sexta*). The larvicidal effects of pericarp extract on egg hatching, caterpillar mass, caterpillar mass gain, and resulted developmental delay evidently indicate their potential candidacy to manage a number of crop insect pests.

## Supplementary Materials

The supplementary materials are available online at https://www.mdpi.com/2075-4450/11/2/98/s1, Table S1: Details of statistical analyses to study the effect of polyphenol rich purple corn pericarp extract on egg hatching, growth, and development of tobacco hornworm; Table S2: Statistical analyses for mass of tobacco hornworm; Table S3: Statistical analyses for mass gain w.r.t. previous mass; Table S4: Statistical analyses for mass gain w.r.t. first instar; Table S5: Statistical analyses for feeding preference; Table S6: Statistical test for larval percent survival; Table S7: Statistical test for the effects of polyphenol-rich purple corn pericarp extract diet switch on mean larval mass; Video S6: A video of fifth instar *M. sexta* caterpillar wandering in the petri plate indicates ready for pupation, Video S7: Ethovision choice assay videos demonstrates the feeding preference of *Manduca sexta* caterpillars for control diet over purple corn pericarp extract diet.
