# Supplementary material for: Polyphenol-Rich Purple Corn Pericarp Extract Adversely Impacts Herbivore Growth and Development
Source: Insects. 2020 Feb 2;11(2):98. doi: 10.3390/insects11020098 (PMC7074539; doi:10.3390/insects11020098)
Supplement: Supplementary file 1 [file insects-11-00098-s001.pdf]

Supplementary Data

Polyphenol-Rich Purple Corn Pericarp Extract Adversely Impacts Herbivore Growth and Development

Mandeep Tayal [1], Pavel Somavat [2], Isabella Rodriguez [3], Tina Thomas [4], Bradley Christoffersen [1] and Rupesh Kariyat [1,*]

[1] Department of Biology, The University of Texas Rio Grande Valley, Edinburg, TX 78539, USA; mandeep.tayal01@utrgv.edu (M.T.); Bradley.christoffersen@utrgv.edu (B.C.)
[2] School of Earth, Environmental, and Marine Sciences, The University of Texas Rio Grande Valley, Edinburg, TX 78539, USA; Pavel.somavat@utrgv.edu
[3] Mathematics and Science Academy, The University of Texas Rio Grande Valley, Edinburg, TX 78539, USA; isabella.rodriguez01@utrgv.edu
[4] Department of Chemistry, The University of Texas Rio Grande Valley, Edinburg, TX 78539, USA; tina.thomas@utrgv.edu
* Correspondence: Rupesh.kariyat@utrgv.edu

Received: 12 January 2020; Accepted: 30 January 2020; Published: date

Table S1. Details of statistical tests used to study the effect of polyphenol-rich purple corn pericarp extract on egg hatching, growth, and development of tobacco hornworm (*M. sexta*). Significant results with *p* values < 0.05 are in bold.

Parameter	Test	Test Statistics	*p*-value
Total anthocyanins	Mann–Whitney Test	Mann–Whitney U=0	**0.0022**
Total tannins	t test	t statistic = 33.33	**<0.0001**
Total polyphenols	Mann–Whitney Tsest	Mann–Whitney U=0	**0.0022**
Egg hatching	Kruskal–Wallis Test	Kruskal–Wallis Statistic = 26.04	**<0.0001**
First instar survival	Kruskal–Wallis Test	Kruskal–Wallis Statistic = 3.756	0.1529
Larval survival	Kruskal–Wallis Test	Kruskal–Wallis Statistic = 6.557	**0.0377**
Time to pupate	Kruskal–Wallis Test	Kruskal–Wallis Statistic = 50.91	**<0.0001**
Ethovision	t-test	t statistic = 0.3063	0.7629
Diet switch (larval mass)	One-way ANOVA	F value = 4.740	**0.0226**
Larval mass gain spray	Kruskal–Wallis Test	Kruskal–Wallis Statistic = 10.54	**0.0051**
Larval frass after spray	One-way ANOVA	F = 5.414	**0.014**

Table S2. Details of statistical tests used to study the effect of polyphenol-rich purple corn pericarp extract on mass of tobacco hornworm (*Manduca sexta* L.). Significant results with *p* values < 0.05 are in bold.

Parameter	Test	Test Statistics	df	*p*-value
Mass 1	Kruskal–Wallis Test	Kruskal–Wallis Statistic = 1.951	2	0.377
Mass 2	Kruskal–Wallis Test	Kruskal–Wallis Statistic = 6.192	2	**0.0452**
Mass 3	Kruskal–Wallis Test	Kruskal–Wallis Statistic = 24.23	2	**<0.0001**
Mass 4	Kruskal–Wallis Test	Kruskal–Wallis Statistic = 7.638	2	**0.022**
Mass 5	Kruskal–Wallis Test	Kruskal–Wallis Statistic = 25.06	2	**<0.0001**
Mass 6	Kruskal–Wallis Test	Kruskal–Wallis Statistic = 34.05	2	**<0.0001**
Mass 7	Kruskal–Wallis Test	Kruskal–Wallis Statistic = 37.87	2	**<0.0001**
Mass 8	Kruskal–Wallis Test	Kruskal–Wallis Statistic = 16.7	2	**0.0002**

Table S3. Details of statistical tests used to study the effect of polyphenol-rich purple corn pericarp extract on mass gain w.r.t. previous mass of tobacco hornworm (*Manduca sexta* L.). Significant results with *p* values < 0.05 are in bold.

Parameter	Test	Test Statistics	df	*p*-value
Mass gain 1	Kruskal–Wallis Test	Kruskal–Wallis Statistic = 4.264	2	0.1186
Mass gain 2	Kruskal–Wallis Test	Kruskal–Wallis Statistic = 15.74	2	**0.0004**
Mass gain 3	Kruskal–Wallis Test	Kruskal–Wallis Statistic = 53.94	2	**<0.0001**
Mass gain 4	Kruskal–Wallis Test	Kruskal–Wallis Statistic = 5.744	2	0.0566
Mass gain 5	Kruskal–Wallis Test	Kruskal–Wallis Statistic = 23.86	2	**<0.0001**
Mass gain 6	Kruskal–Wallis Test	Kruskal–Wallis Statistic = 1.398	2	0.4971
Mass gain 7	Kruskal–Wallis Test	Kruskal–Wallis Statistic = 21.5	2	**<0.0001**
Mass gain 8	Kruskal–Wallis Test	Kruskal–Wallis Statistic = 14.88	2	**0.0006**

Table S4. Details of statistical tests used to study the effect of polyphenol-rich purple corn pericarp extract on mass gain w.r.t. first instar of tobacco hornworm (*Manduca sexta* L.). Significant results with *p* values < 0.05 are in bold.

Parameter	Test	Test Statistics	df	*p*-value
Mass gain 1	Kruskal–Wallis Test	Kruskal–Wallis Statistic = 4.264	2	0.1194
Mass gain 2	Kruskal–Wallis Test	Kruskal–Wallis Statistic = 15.74	2	**0.0028**
Mass gain 3	Kruskal–Wallis Test	Kruskal–Wallis Statistic = 53.94	2	**<0.0001**
Mass gain 4	Kruskal–Wallis Test	Kruskal–Wallis Statistic = 5.744	2	**0.0002**
Mass gain 5	Kruskal–Wallis Test	Kruskal–Wallis Statistic = 23.86	2	**<0.0001**
Mass gain 6	Kruskal–Wallis Test	Kruskal–Wallis Statistic = 1.398	2	**<0.0001**
Mass gain 7	Kruskal–Wallis Test	Kruskal–Wallis Statistic = 21.5	2	**<0.0001**
Mass gain 8	Kruskal–Wallis Test	Kruskal–Wallis Statistic = 14.88	2	**0.0001**

Table S5. Details of statistical analyses used to study the effect of polyphenol-rich purple corn pericarp extract on feeding preference of tobacco hornworm (*Manduca sexta* L.) caterpillars. Significant results with *p* values < 0.05 are in bold.

Parameter	Test	Test Statistics	df	*p*-value
On/Off 1	Kruskal–Wallis Test	Kruskal–Wallis Statistic = 26.49	2	**<0.0001**
On/Off 2	Kruskal–Wallis Test	Kruskal–Wallis Statistic = 8.335	2	**0.0155**
On/Off 3	Kruskal–Wallis Test	Kruskal–Wallis Statistic = 21.03	2	**<0.0001**
On/Off 4	Kruskal–Wallis Test	Kruskal–Wallis Statistic = 17.53	2	**0.0002**
On/Off 5	Kruskal–Wallis Test	Kruskal–Wallis Statistic = 10.27	2	**0.0059**
On/Off 6	Kruskal–Wallis Test	Kruskal–Wallis Statistic = 7.859	2	**0.0196**
On/Off 7	Kruskal–Wallis Test	Kruskal–Wallis Statistic = 2.987	2	0.2245
On/Off 8	Kruskal–Wallis Test	Kruskal–Wallis Statistic = 12.07	2	**0.0024**

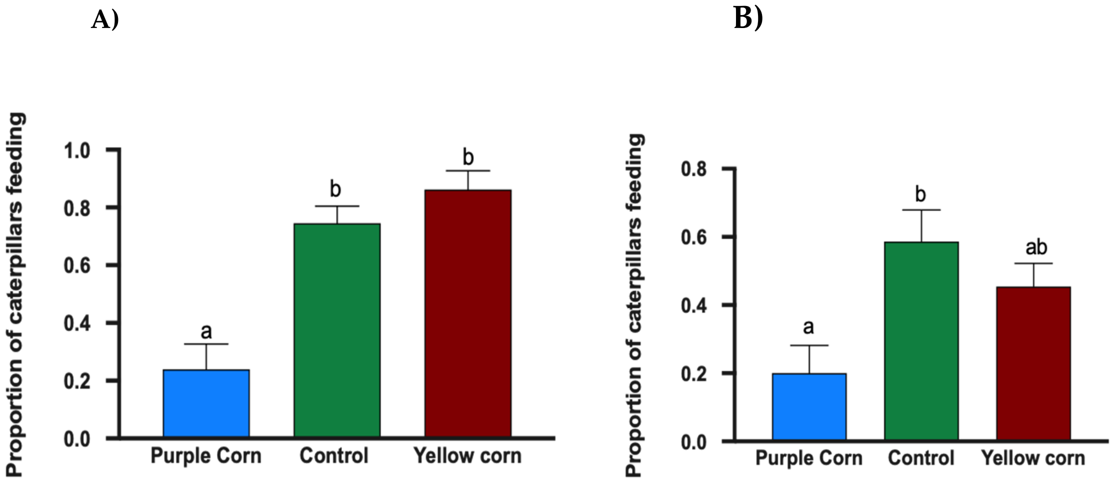

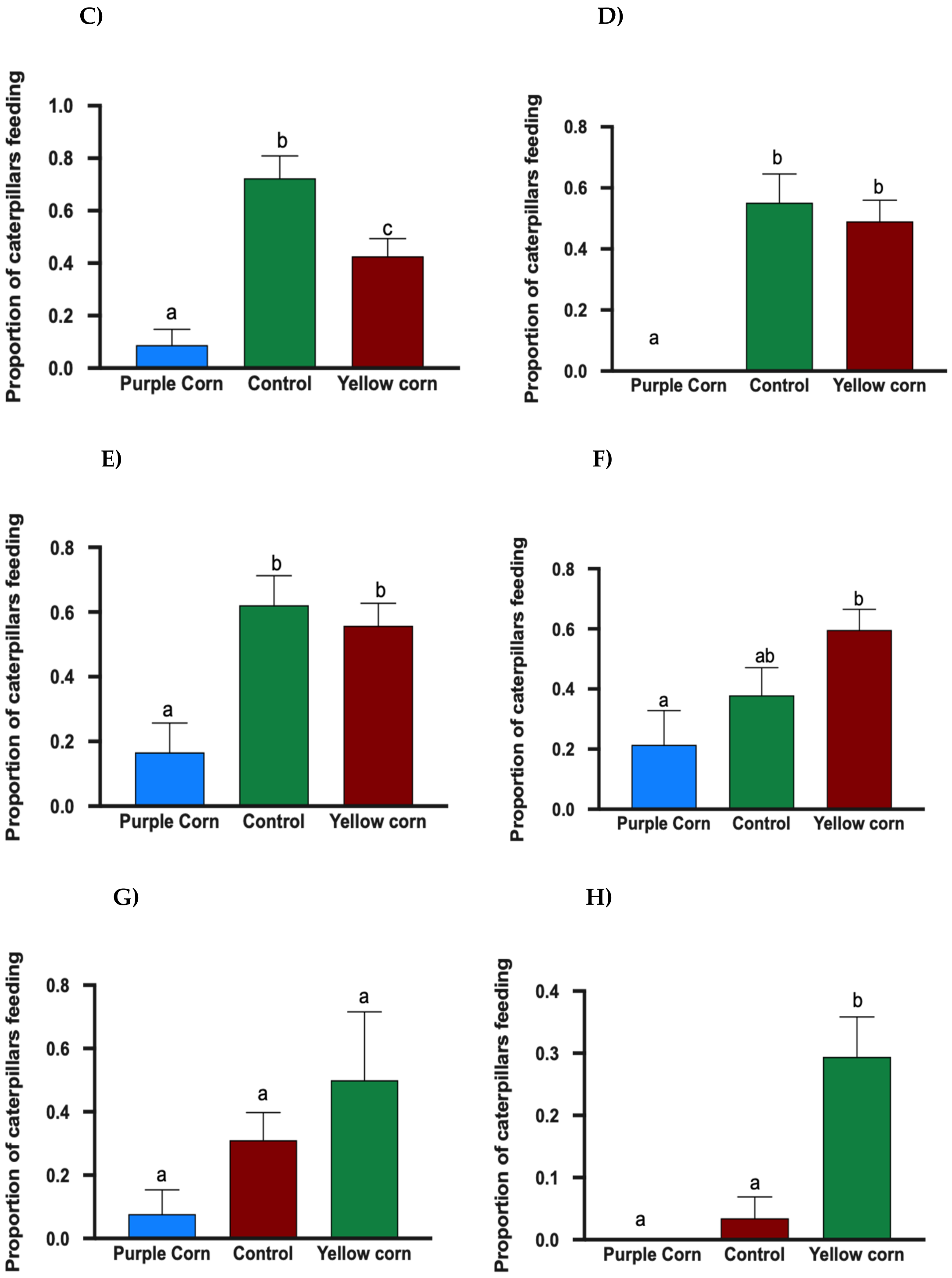

Figure S1. Proportion of larval feeding preference of eight observations (A-H) among different treatments recorded throughout larval stages. Means followed by same different letters are not statistically significantly different (Kruskal–Wallis tests, Dunn's multiple comparison test, at $p < 0.05$).

Table S6. Details of statistical analysis used to study the effect of polyphenol-rich purple corn pericarp extract on percent larval survival of tobacco hornworm (*Manduca sexta* L.). Significant results with *p* values <0.05 are in bold.

Parameter	Test	Test Statistics	df	*p*-value
Percent survival	Log-rank (Mantel–Cox) test	Chi-square = 2.13	2	**<0.3447**

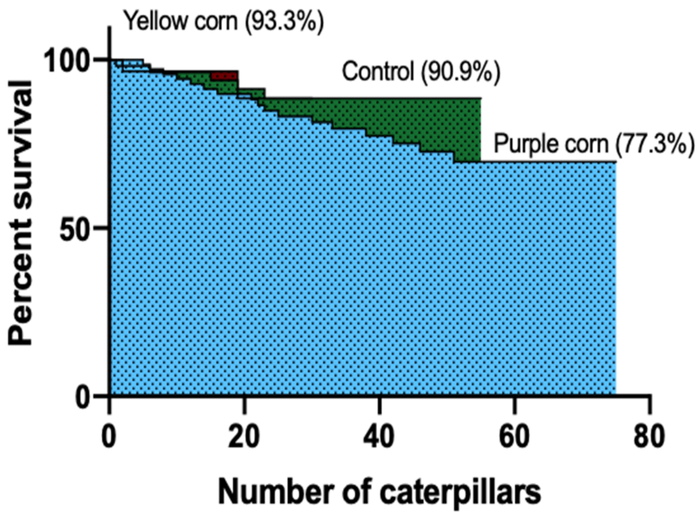

Figure S2. Percent survival of caterpillars among different treatments throughout larval stages (Log-rank (Mantel–Cox) test, $p < 0.05$).

Table S7. Details of statistical tests for the effects of polyphenol rich purple corn pericarp extract diet switch on mean larval mass of *M. sexta*. Significant results with p values < 0.05 are in bold.

Parameter	Test	Test Statistics	df	*p*-value
Larval mass 1 before switch	Unpaired t-test	t = 1.545	df = 36	*p* = 0.1311
Larval mass 2 before switch	Unpaired t-test	t = 2.226	df = 33	***p* = 0.0329**
Larval mass 1 after switch	ANOVA	F value = 3.445	df = 2	*p* = 0.0508
Larval mass 2 after switch	ANOVA	F value = 4.740	df = 2	***p* = 0.0200**

Supplementary Video S6

https://utrgv-my.sharepoint.com/:f:/g/personal/mandeep_tayal01_utrgv_edu/EnoqqR-AJZdLoxFt8obtAkkBUZxwgGMgCyQfPk119X9LxA?e=bcxh5T

Supplementary Videos S7

A link to the Ethovision choice assay videos demonstrates the feeding preference of *Manduca sexta* caterpillars for control diet over purple corn pericarp extract diet.

https://www.dropbox.com/sh/guo2p4qd6202k2e/AAAmhOS4ucDlQfpEDdWnh_q8a?dl=0

© 2020 by the authors. Submitted for possible open access publication under the terms and conditions of the Creative Commons Attribution (CC BY) license (http://creativecommons.org/licenses/by/4.0/).
